# Acetyl-CoA Deficiency Is Involved in the Regulation of Iron Overload on Lipid Metabolism in Apolipoprotein E Knockout Mice

**DOI:** 10.3390/molecules27154966

**Published:** 2022-08-04

**Authors:** Gang Luo, Lu Xiang, Lin Xiao

**Affiliations:** 1 Department of Health Toxicology, Xiangya School of Public Health, Central South University, Changsha 410078, China; 2 Department of Nutrition Science and Food Hygiene, Xiangya School of Public Health, Central South University, Changsha 410078, China

**Keywords:** iron overload, acetyl-CoA, non-alcoholic fatty liver disease, cholesterol, proteomics, oxidative stress

## Abstract

The role of dietary iron supplementation in the development of nonalcoholic fatty liver disease (NAFLD) remains controversial. This study aimed to investigate the effect of excess dietary iron on NAFLD development and the underlying mechanism. Apolipoprotein E knockout mice were fed a chow diet, a high-fat diet (HFD), or an HFD containing 2% carbonyl iron (HFD + Fe) for 16 weeks. The serum and liver samples were acquired for biochemical and histopathological examinations. Isobaric tags for relative and absolute quantitation were performed to identify differentially expressed proteins in different groups. Excess dietary iron alleviated HFD-induced NAFLD, as evidenced by significant decreases in serum/the hepatic accumulation of lipids and the NAFLD scores in HFD + Fe-fed mice compared with those in HFD-fed mice. The hepatic acetyl-CoA level was markedly decreased in the HFD + Fe group compared with that in the HFD group. Important enzymes involved in the source and destination of acetyl-CoA were differentially expressed between the HFD and HFD + Fe groups, including the enzymes associated with cholesterol metabolism, glycolysis, and the tricarboxylic acid cycle. Furthermore, iron overload-induced mitochondrial dysfunction and oxidative stress occurred in mouse liver, as evidenced by decreases in the mitochondrial membrane potential and antioxidant expression. Therefore, iron overload regulates lipid metabolism by leading to an acetyl-CoA shortage that reduces cholesterol biosynthesis and might play a role in NAFLD pathogenesis. Iron overload-induced oxidative stress and mitochondrial dysfunction may impair acetyl-CoA formation from pyruvate and β-oxidation. Our study provides acetyl-CoA as a novel perspective for investigating the pathogenesis of NAFLD.

## 1. Introduction

Nonalcoholic fatty liver disease (NAFLD) is the most common chronic liver disease worldwide, affecting approximately 25% of the population [1]. NAFLD encompasses a spectrum of hepatic histopathological changes, including isolated steatosis, nonalcoholic steatohepatitis, and subsequent fibrosis and cirrhosis [2]. It is strongly associated with metabolic and cardiovascular disorders, of which cardiovascular disease is the leading cause of death in NAFLD patients [3]. The cardinal feature of NAFLD is the excess hepatic accumulation of lipids, including triglycerides, free fatty acids, free cholesterol, and other lipid metabolites, in the absence of significant alcohol consumption [4]. The disturbance of lipid homeostasis results from an imbalance between lipid acquisition and disposal, i.e., the hepatic lipid uptake and de novo lipogenesis exceeding fatty acid oxidation and lipid export [5,6].

Acetyl-CoA plays a key role in lipid homeostasis by interacting with different metabolic pathways and transformations [7]. The synthesis of acetyl-CoA is the rate-limiting process in cholesterol synthesis [7], and cholesterol synthesis is involved in the assembly of lipoproteins for delivery to peripheral tissues [8]. In the liver, the mitochondrial pyruvate dehydrogenase (PDH) complex catalyzes pyruvate generated from glycolysis to produce acetyl-CoA [7]. Acetyl-CoA is also produced through fatty acid β-oxidation and amino acid catabolism. In the mitochondria, acetyl-CoA is oxidized via the tricarboxylic acid (TCA) cycle to generate ATP or ketone bodies through the hydroxymethylglutaryl-CoA system or to carnitine/acetyl-CoA that exits from the mitochondria to the cytosol. In the cytosol, carnitine/acetyl-CoA is converted to acetyl-CoA to initiate cholesterol or fatty acid synthesis [9]. Thus, acetyl-CoA fluctuations within subcellular compartments might determine the metabolic routes of lipids, playing an important role in the pathogenesis of NAFLD.

Iron overload appears to aggravate NAFLD, as evidenced by mild hepatic iron deposition and elevations in serum ferritin with normal or mildly elevated transferrin saturation in one-third of patients with NAFLD [10]. Oxidative stress and apoptosis are involved in the progression of NAFLD [11], and iron overload has been shown to increase hepatic oxidative stress and apoptosis in animal models and patients with NAFLD [12,13]. On the contrary, some studies have shown that iron deficiency is positively associated with obesity and hepatic steatosis [14,15]. Furthermore, iron deficiency may increase hepatic lipogenesis and deteriorate systemic lipid homeostasis [16,17]. Changes in iron metabolism are associated with changes in lipid pathways, including cholesterol, sphingolipids, and lipid droplets [18]. We have previously found that dietary iron overload mitigates atherosclerosis in high-fat diet (HFD)-fed apolipoprotein E knockout (ApoE-KO) mice by impairing hepatic fatty acid metabolism [19]. Based on the above observations, we hypothesized that dietary iron overload might play a role in NAFLD pathogenesis by impairing hepatic lipid metabolism and that acetyl-CoA could play an important role in this process.

To test our hypothesis, we explored the effect of excess dietary iron on lipid metabolites and identified differentially expressed proteins related to the metabolic routes of acetyl-CoA in a NAFLD mouse model. Our study, for the first time, suggests that acetyl-CoA deficiency is involved in the regulation of iron overload on lipid metabolism. It also suggests acetyl-CoA fluctuation as a new perspective for investigating the pathogenesis of NAFLD.

## 2. Materials and Methods

### 2.1. Animals and Treatment

Animals were treated following the Guiding Principles in the Care and Use of Animals approved by the Animal Care and Use Committee of the Central South University (Changsha, Hunan, China) (approval #XYGW-2021-82). Six-week-old female ApoE KO mice (18–20 g) with a C57BL/6J genetic background (*n* = 36) were obtained from Beijing Vital River Laboratory Animal Technology Co., Ltd. (Beijing, China). Sex differences have been frequently reported in mouse atherosclerosis studies using ApoE KO mice with a C57BL/6J genetic background [20]. The most widely reported sex effect on atherosclerosis is that female ApoE KO mice exhibit mature fibrofatty atherosclerotic plaques as early as 16 weeks of age, and they have significantly larger lesions compared with age-matched males [21,22]. Therefore, only female ApoE mice were used in the present study. After one week of acclimatization with a chow diet and water *ad libitum*, the mice were randomly divided into three groups (*n* = 12 per group) and fed a normal chow diet (ND), HFD (an atherogenic diet containing 21% fat and 0.15% cholesterol), or HFD supplemented with 2% carbonyl iron (HFD + Fe) for 16 weeks. All animals were maintained under a specific pathogen-free environment in a standard light/darkness cycle (12 h/12 h) with free access to food and water.

### 2.2. Sample Collection and Histological Examination

After 16 weeks of diet intervention, four mice were randomly selected from each group for en face aorta oil red O staining as previously described [23]. After overnight fasting, the remaining animals were euthanized. The livers were harvested, embedded in optimal cutting temperature compound, frozen on dry ice, and then stored at −80 °C until sectioning. Hepatic tissue was cut into 8-mm-thick slices for hematoxylin and eosin (H&E) staining, 4-Hydroxy-2-nonenal (4HNE) immunohistochemical staining, or Prussian blue staining. The NAFLD activity score was assessed as previously described [24].

### 2.3. Measurements of Metabolites

Liver samples were homogenized in phosphatase buffered saline solution. Hepatic and serum levels of cholesterol, high-density lipoprotein cholesterol (HDL-C), and LDL-C were measured using commercial kits from BioSino (Beijing, China) according to the manufacturer’s instructions. Lactic acid, iron, superoxide dismutase (SOD), and reduced glutathione (GSH) serum levels were determined using the kits from Nanjing Jiancheng Bioengineering Institute (Nanjing, China). Liver iron concentration was measured using flame atomic absorption spectrometry (SpectrAA240FS; Palo Alto, CA, USA). The normal value of female mouse liver iron is 222–658 µg/g protein [25]. In the iron overload model, the liver iron levels and serum iron levels in the model group are usually significantly higher than those in the control group, indicating that the modeling is successful. Hepatic ATP and acetyl-CoA levels were measured using the kits from Sigma-Aldrich (#MAK190, #MAK 039; Saint Louis, MO, USA) following the manufacturer’s protocol.

### 2.4. Mitochondrial Membrane Potential (MMP)

Mitochondria were extracted from fresh liver tissue using differential centrifugation [26]. MMP was measured using fluorescent dye 5,5′6,6′-tetrachloro-1,1′3,3′-tetraethylbenzimidazol-carbocyanine iodide (Beyotime Institute of Biotechnology, Shanghai, China).

### 2.5. Isobaric Tags for Relative and Absolute Quantitation (iTRAQ)

Total proteins were isolated from liver tissue samples (*n* = 3/group) for iTRAQ proteomics analysis as previously described [19]. Briefly, the protein samples were reduced using 10 mM DTT and then alkylated with 55 mM IAM, followed by precipitation in cold acetone for 3 h at −20 °C and centrifugation at 20,000× *g* for 30 min. The precipitates were resuspended in a buffer solution containing 50% TEAB and 0.1% SDS. Then, 100 μg proteins were digested using 1 μg/μL trypsin overnight at 37 °C. The peptides were labeled with 117 (ND group), 118 (HFD group), and 121 (HFD + Fe group) iTRAQ tags. LC-MS/MS analysis was performed using an Easy Nano liquid chromatography apparatus system coupled to a Q-Exactive mass spectrometer (Thermo Fisher Scientific, Sunnyvale, CA, USA) as previously described [19].

### 2.6. Protein Identification and Quantification

Protein identification and quantification were performed using ProteinPilot v.4.5 (AB SCIEX). The proteins were searched against the International SwissProt (Mouse) database (http://www.expasy.org, access date, 22 November 2021). The relative quantification analysis of peak areas of the reporter ions was performed to obtain the total proteins for each group with a relative ratio. The protein ratio was calculated by averaging the ratios of three replicates. *p*-values < 0.05 and fold change > 1.5 (upregulation) or <0.67 (downregulation) were used as cut-off values to define differentially expressed proteins.

### 2.7. Quantitative Real-Time PCR (qRT-PCR)

Total RNA was extracted from mouse liver samples using TRIzol (Thermo Fisher Scientific, Sunnyvale, CA, USA). qRT-PCR was performed using SYBR green (TaKaRa Bio, Dalian, China) on an Applied Biosystems 7900 HT PCR instrument (Applied Biosystems, Waltham, MA, USA). Data were analyzed using the comparative 2 ^−ΔΔCt^ method normalized to β-actin. The primer sequences are summarized in Table 1.

### 2.8. Western Blot Analysis

Western blotting was performed as previously described [23]. Briefly, protein concentration was determined using a BCA kit (Beyotime Institute of Biotechnology, Shanghai, China). Protein samples were separated using 10% SDS-PAGE gel and transferred onto a PVDF membrane (MilliporeSigma, Burlington, MA, USA) for 2 h at 300 mA. After blocking, the membranes were incubated with primary antibody against squalene monooxygenase (SQLE) (12544-1-AP; Proteintech, Rosemont, IL, USA), hydroxy-3-methylglutaryl coenzyme A reductase (HMGCR) (bs-5068R; Bioss, Woburn, MA, USA), HMGCS1 (ab42201; Abcam, Cambridge, UK), pyruvate dehydrogenase E1 component subunit beta (PDHB) (ab155996; Abcam), isocitrate dehydrogenase 3A (IDH3a) (ab154886; Abcam), glucokinase (GCK) (ab88056; Abcam), or 4-hydroxynonenal (4HNE) (ab46545; Abcam) overnight at 4 °C, followed by incubation with horseradish peroxidase-conjugated secondary antibody for 1 h at room temperature. Immunoreactivity was detected using an ECL detection system (Amersham Biosciences, Little Chalford, UK).

### 2.9. Statistical Analysis

Data were expressed as the mean ± standard deviation. Statistical analysis was performed using SPSS 18.0 software (IBM, Armonk, NY, USA). The statistical significance between groups was analyzed by Student’s *t*-test or one-way analysis of variance. A value of *p* < 0.05 was considered statistically significant.

## 3. Results

### 3.1. Dietary Iron Overload Alleviates HFD-Induced NAFLD in ApoE KO Mice

**Figure 1:**
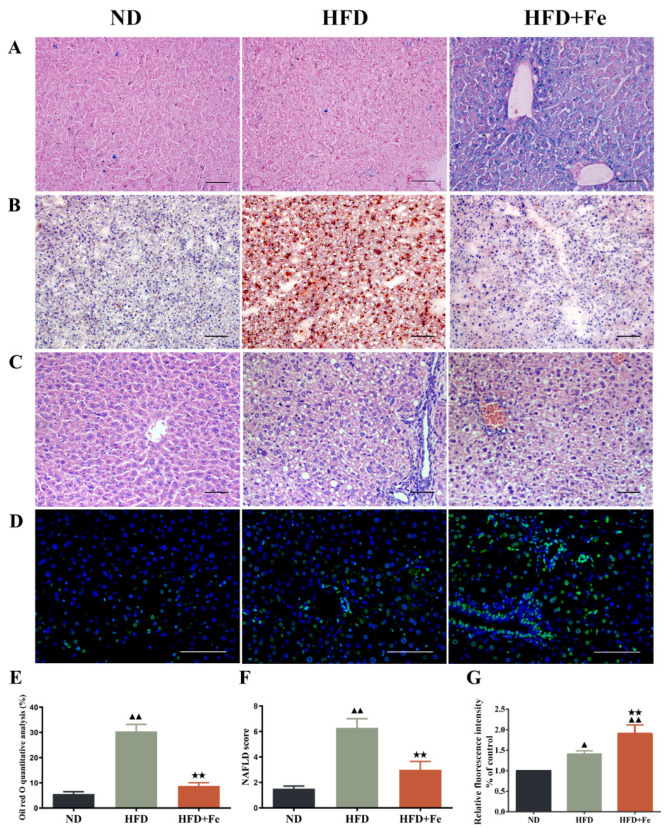
Histopathological observation of mouse liver. Six-week-old female ApoE knockout C57BL/6J mice (18–20 g) were randomly divided into three groups (*n* = 12 per group) and fed a normal chow diet (ND), high-fat diet (HFD), or HFD supplemented with 2% carbonyl iron (HFD + Fe) for 16 weeks. The liver tissue samples were collected from 4 mice randomly selected from each group and subjected to *Prussian blue* staining (**A**), Oil red O staining (**B**), H&E staining (**C**), and TUNEL staining (**D**). (**E**) Quantification of B. (**F**) NAFLD scores based on H&E staining. (**G**) Quantification of (**D**). Scale bar: 50 μm. Data were expressed as the mean ± standard deviation (SD). ^▲^ *p* < 0.05 vs. ND; ^▲▲^ *p* < 0.01 vs. ND; ^★★^ *p* < 0.01 vs. HFD. *n* = 4. ND: normal diet; HFD: high-fat diet, HFD + Fe: high-fat diet supplemented with 2% carbonyl iron.

To explore the effect of excess iron intake on HFD-induced NAFLD, we fed ApoE KO mice with HFD with or without excess iron supplements for 16 weeks. Prussian blue staining confirmed the hepatic iron deposition in HFD + Fe-fed mice (Figure 1A). Consistently, the serum iron level was markedly increased in HFD + Fe group compared with that in ND or HFD group, and the serum hepcidin levels were higher in the HFD + Fe group than in the ND and HFD groups (Table 2). HFD had no impact on the ferritin light and heavy chains, but the HFD + Fe group showed higher levels of ferritin light and heavy chains (Supplementary file Figure S1 and Table S1). Oil red-O staining and H&E staining showed that HFD resulted in dramatic increases in lipid accumulation and NAFLD scores in the liver compared with ND. Importantly, HFD-induced effects were abolished by HFD + Fe feeding (Figure 1B,C,E,F). Meanwhile, HFD-induced elevations in serum cholesterol, serum LDL-C, and hepatic cholesterol levels were effectively reversed by iron supplementation in HFD (Table 2). Despite significantly enhanced TUNEL signals in the liver and increased serum AST and ALT levels in HFD + Fe-fed mice compared with those in HFD-fed mice (Figure 1D,G, Table 2), our data suggest that iron overload affects lipid metabolism, which might affect the development of NAFLD. The results showed that compared with the ND group, there was no significant difference in the expression levels of serum hepcidin and liver ferritin light and heavy chains, indicating that HFD did not significantly affect liver iron metabolism. However, compared with the HFD group, the serum hepcidin and ferritin light and heavy chain levels were significantly increased in the HFD + Fe group, and there was an obvious iron overload phenomenon.

### 3.2. Excess Dietary Iron Reverses HFD-Induced Elevation in Hepatic Acetyl-CoA Level in ApoE KO Mice

**Figure 2:**
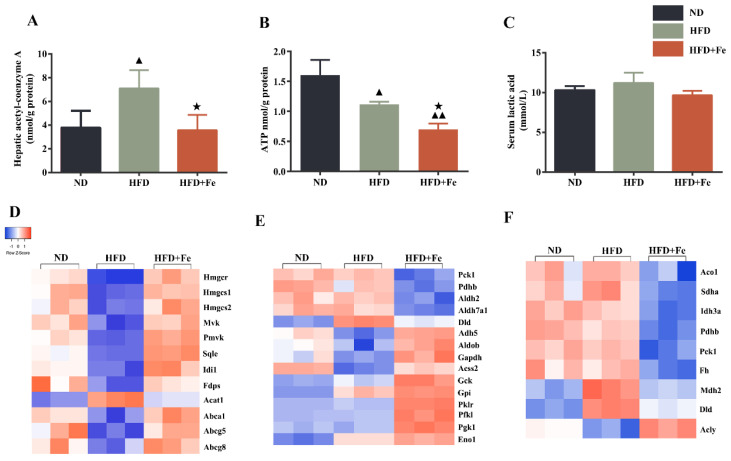
Dietary iron overload-induced metabolic changes in ApoE KO mice. (**A**–**C**) Hepatic acetyl-CoA and ATP levels and serum lactic acid content were determined at 16 weeks after diet intervention. Data were expressed as the mean ± SD. ^▲^ *p* < 0.05 vs. ND, ^▲▲^ *p* < 0.01 vs. ND, ^★^ *p* < 0.05 vs. HFD; *n* = 6. (**D**–**F**) Heatmap of differentially expressed hepatic proteins involved in cholesterol metabolism (**D**), glycolysis (**E**), and tricarboxylic acid (TCA) cycle (**F**). ND: normal diet; HFD: high-fat diet, HFD + Fe: HFD supplemented with 2% carbonyl iron.

NAFLD is characterized by the hepatic accumulation of free fatty acid and cholesterol [27]. Because cholesterol synthesis generally takes place in the liver and begins from acetyl-CoA [28], we determined the hepatic acetyl-CoA concentration in mice. We found that compared with that in ND-fed mice, the hepatic acetyl-CoA concentration was significantly elevated in HFD-fed mice but remained unchanged in HFD + Fe-fed mice (Figure 2A), suggesting that acetyl-CoA deficiency in the presence of excess iron might be responsible for the reduced serum and hepatic cholesterol levels in HFD + Fe-fed mice. We also noticed a significant reduction in hepatic ATP production in HFD + Fe-fed mice compared with that in HFD-fed mice (Figure 2B), suggesting a decreased TCA cycle due to a shortage of acetyl-CoA and/or mitochondrial dysfunction in the presence of excess iron. No significant change in lactic acid serum level was observed among different groups (Figure 2C). These results suggest that excess dietary iron abrogates the promoting effect of HFD on hepatic acetyl-CoA generation, thus reducing cholesterol biosynthesis and the TCA cycle in the liver.

### 3.3. The Proteins Related to Cholesterol Metabolism, Glycolysis, and the TCA Cycle Are Differentially Expressed between HFD + Fe-fed Mice and HFD-fed Mice

To identify the proteins that are potentially involved in acetyl-CoA deficiency in the liver of mice exposed to excess iron, we performed a proteomics analysis. We found that proteins related to cholesterol metabolism (Figure 2D), glycolysis (Figure 2E), and the TCA cycle (Figure 2F) exhibited differential and converse expression patterns between the HFD and HFD + Fe groups (Table S1). In the differentially expressed proteins related to cholesterol metabolism, we noticed that the expression of acetyl-CoA acetyltransferase 1 (ACAT1), which catalyzes the formation of acetoacetyl-CoA from two acetyl-CoA to initiate cholesterol biosynthesis [29], was noticeably downregulated in the HFD + Fe group compared with that in the HFD group. In addition, the ABC family members ABCA1, ABCG5, and ABCG8, which pump the cholesterol out of cells [30], were significantly upregulated in the HFD + Fe group compared with those in the HFD group, reaching comparable levels to those in the ND group. These data suggest that HFD + Fe intervention alleviates NAFLD, possibly by suppressing cholesterol formation and facilitating cholesterol efflux through altering the expression of corresponding enzymes.

**Figure 3:**
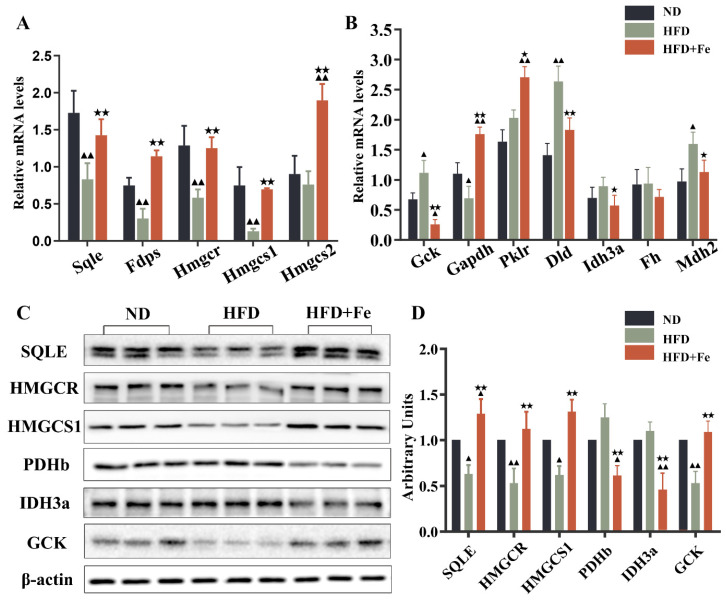
Validation of alterations in differentially expressed proteins. Quantitative real-time PCR (**A**,**B**) and Western blot analysis (**C**) were conducted to validate the alterations in representative differentially expressed proteins identified by proteomics analysis. (**D**) Quantification of (**C**). Data were expressed as the mean ± SD. ^▲^ *p* < 0.05, ^▲▲^ *p* < 0.01 vs. ND; ^★^ *p* < 0.05, ^★★^ *p* < 0.01 vs. HFD. *n* = 4–6. ND: normal diet; HFD: high-fat diet, HFD + Fe: HFD supplemented with 2% carbonyl iron.

In the differentially expressed proteins related to glycolysis (Figure 2E), PDHB is the component of the PDH complex that catalyzes the formation of acetyl-CoA from pyruvate. We found that PDHB expression was markedly repressed in the HFD + Fe group compared with that in the HFD group. In addition, the proteins related to the TCA cycle were generally downregulated in the HFD + Fe group compared with those in the HFD group (Figure 2F), including mitochondrial aconitate hydratase, isocitrate dehydrogenase alpha subunit (IDH3A), dihydrolipoyl dehydrogenase, and mitochondrial malate dehydrogenase. Of note, some genes are sometimes involved in multiple pathways, and their involvement depends upon the metabolic conditions. To confirm the results of Figure 2, qRT-PCR and Western blot analysis further showed that HFD + Fe significantly altered the expression of enzymes involved in cholesterol metabolism, glycolysis, and the TCA cycle, including SQLE, HMGCR, HMGCS1, PDHb, IDH3A, and GCK (Figure 3A–D). Taken together, these data suggest that excess dietary iron suppresses acetyl-CoA formation, possibly through downregulating glycolysis, leading to a reduction in cholesterol biosynthesis and thus preventing HFD-induced lipid deposition.

### 3.4. Excess Dietary Iron Induces Hepatic Mitochondrial Dysfunction and Oxidative Damage in ApoE KO Mice

**Figure 4:**
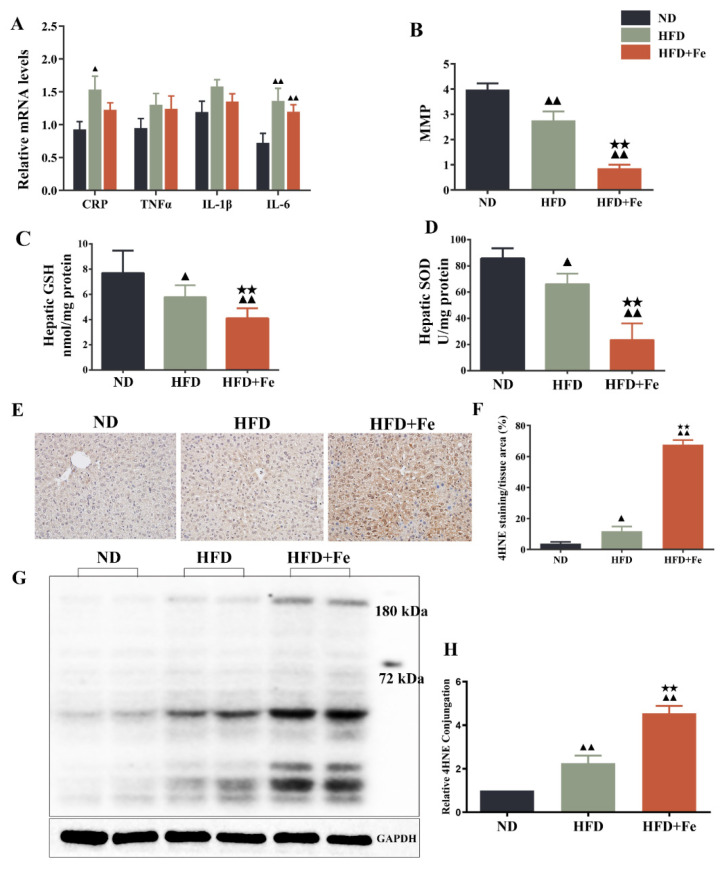
Dietary iron overload-induced hepatic oxidative damage in ApoE KO mice. (**A**) Inflammatory markers. (**B**) Hepatic levels of matrix metalloproteinase (MMP). (**C**,**D**) Hepatic levels of glutathione (GSH) and superoxide dismutase (SOD). (**E**) Immunohistochemical staining of 4-hydroxy-2-nonenal (4HNE). (**F**) Quantification of (**E**). (**G**) Western blot analysis was performed to determine the mitochondrial expression of 4HNE. (**H**) Quantification of (**G**). Data were expressed as the mean ± SD. ^▲^ *p* < 0.05, ^▲▲^ *p* < 0.01 vs. ND; ^★★^ *p* < 0.01 vs. HFD. ND: normal diet; HFD: high-fat diet, HFD + Fe: HFD supplemented with 2% carbonyl iron; MMP: mitochondrial membrane potential; SOD: superoxide dismutase; GSH: glutathione; 4HNE: 4-hydroxy-2-nonenal.

Since acetyl-CoA is mainly derived from glycolysis-produced pyruvate in the mitochondria [7], we speculated that iron-induced acetyl-CoA deficiency might be due to dysfunctional mitochondria. Compared with the HFD group, the HFD + Fe group showed no significant difference in the levels of CRP, TNF-α, IL-1β, and IL-6 (Figure 4A). As shown in Figure 4B–D, HFD significantly reduced MMP and the production of antioxidant GSH and SOD in mouse liver compared with ND. Of note, these effects were further reinforced by HFD + Fe. Meanwhile, HFD + Fe dramatically enhanced the HFD-induced increase in the protein level of the oxidative stress marker 4HNE [31] in the liver tissue samples (Figure 4E,F) and the hepatocyte mitochondria (Figure 4G,H) of mice. These data collectively suggest that excess dietary iron exacerbates HFD-induced hepatic mitochondrial dysfunction and oxidative stress in ApoE KO mice, leading to an impairment in acetyl-CoA synthesis.

## 4. Discussion

In this study, we found that HFD failed to induce NAFLD in ApoE KO mice in the presence of excess iron, possibly due to an acetyl-CoA shortage. Proteomics analysis revealed that excess dietary iron resulted in differential expressions of important proteins involved in acetyl-CoA metabolic routes, including cholesterol metabolism, glycolysis, and the TCA cycle. Furthermore, excess dietary iron impaired mitochondria function and increased oxidative stress in the livers of ApoE KO mice. These findings suggest that acetyl-CoA deficiency is involved in the regulation of iron overload on lipid metabolism. It appeared to slow down the development of NAFLD by impairing cholesterol biosynthesis caused by the acetyl-CoA shortage. Future studies will have to examine fibrosis in relation to iron overload and changes in acetyl-CoA metabolism, especially considering that iron overload increases oxidative stress and mitochondrial dysfunction, which could lead to adverse effects on the liver other than lipid deposition.

Consistent with our results, Kitamura et al. have recently demonstrated that 15 weeks of HFD intake supplemented with 0.023% (*w*/*w*) sodium ferrous citrate iron reduces body weight gain and hepatic lipid accumulation in male C57BL/6J mice. However, they also have shown that iron supplementation reduces mitochondrial abnormalities while increasing the transcription of genes associated with energy metabolism in the liver and skeletal muscle [32], which is contrary to our findings. On the other hand, Choi et al., who have used the same dose of carbonyl iron as ours in a shorter course (7 weeks), have found that HFD + Fe reduces SOD and glutathione peroxidase protein expression in mouse liver, which is consistent with our findings. In addition, compared with the HFD group, the HFD + Fe group showed no significant difference in the levels of CRP, TNF-α, IL-1β, and IL-6, which is consistent with Altamura et al. [33]. However, unlike us, they have observed that iron supplementation increases the accumulation of hepatic fat, along with inflammatory foci [34]. The contradictions among different studies are possibly due to differences in iron dosages and durations as well as the genetic background of the mice.

Dyslipidemia in NAFLD is characterized by hypertriglyceridemia, hypercholesterolemia, increased LDL-C, and decreased HDL-C [35]. In the present study, we found that iron supplementation abolished HFD-induced increases in serum cholesterol and LDL-C levels as well as hepatic cholesterol levels in mice, in line with the reductions in lipid accumulation and NAFLD scores in the HFD + Fe group versus those in the HFD group. Consistent with our findings, Turbino-Ribeiro et al. have reported that iron dextran injection not only reduces plasma cholesterol levels in rats fed a cholesterol diet but also results in a redistribution of cholesterol among the various lipoprotein fractions, with an elevation in HDL-C and a decrease in LDL-C [36]. Moreover, iron dextran injection exerts a hypocholesterolemic effect on the rabbits fed a cholesterol diet, leading to a significant decrease in aortic arch lesion area by 56% compared with controls [37].

To investigate the mechanisms underlying the preventive role of iron overload in NAFLD, we focused on acetyl-CoA, which sits at the interface of multiple metabolic pathways. Our results showed that iron supplementation blocked HFD-induced elevation in hepatic acetyl-CoA concentration in ApoE KO mice, suggesting that the reduced serum and hepatic lipid accumulation in HFD + Fe-fed mice is possibly due to insufficient acetyl-CoA. As revealed by proteomics analysis, the decreased lipid accumulation in the presence of excess iron is also possibly due to the downregulation of ACAT1 that initiates cholesterol biosynthesis [29] and the upregulation of the ABC family members that pump the cholesterol out of cells [30] in the HFD + Fe group. In the meantime, cholesterol biosynthesis-related enzymes, including HMGCS1/2, HMGCR, MVK, PMK, IDI1, FPPS, and SQLE, were significantly upregulated in the HFD + Fe group compared with those in the HFD group. Similar results have been observed by Graham et al. [38]. This paradox is possibly due to compensation in response to an acetyl-CoA shortage.

As indicated by proteomics analysis, the expression of PDHB that catalyzes the formation of acetyl-CoA from pyruvate was markedly attenuated in the HFD + Fe group compared with that in the HFD group. This may explain the acetyl-CoA deficiency in response to iron supplementation. In addition, the general decreases in protein levels of the TCA cycle-related enzymes in the HFD + Fe group further imply the shortage of acetyl-CoA. Thus, dietary iron overload in ApoE KO mice might prevent the accumulation of cholesterol and the subsequent development of NAFLD due to the lack of substrate for cholesterol biosynthesis in the liver.

It is well established that iron overload causes mitochondrial oxidative damage and mitochondrial dysfunction [39,40]. We also observed that the expressions of the TCA cycle-related enzymes were significantly downregulated in the presence of excess iron, accompanied by a reduction in ATP production, suggesting mitochondria dysfunction in response to excess iron. The remarkable decreases in MMP and the hepatic levels of GSH and SOD, along with a substantial increase in 4HNE protein expression, further confirm that iron overload induces hepatic mitochondrial oxidative stress.

In this study, CH synthesis was reduced in FE + HFD mice, HMGCR gene expression was greater in the FE + HFD group than in HFD-fed mice, and the protein expression of HMGCR and SQLE in the FE + HFD group was greater than in the ND group. The high expression of cholesterol synthesis-related proteins is to compensate for the reduction in cholesterol levels caused by insufficient acetyl-CoA, the precursor for cholesterol synthesis. Therefore, insufficient precursors will lead to a compensatory mechanism being activated to upregulate the synthesis-related proteins to try to increase the cholesterol levels, but due to a serious shortage of precursors, cholesterol levels will appear to decrease. Considering that iron overload-induced oxidative stress and mitochondrial dysfunction might impair acetyl-CoA formation from pyruvate and β-oxidation, it is proposed that hepatic cholesterol biosynthesis was enhanced to compensate for the acetyl-CoA deficiency in dietary iron-overloaded ApoE KO mice.

This study has some limitations. First, the effect of iron overload on lipid metabolism seems dose-, duration-, and genetic background-dependent. These issues need to be addressed in future studies. Second, our study focused on the effect of iron on HFD-induced NAFLD, and an ND + Fe group is absent. The effect of iron supplementation on other diets needs to be investigated in the future. Finally, even though the results might suggest the benefits of iron overload against the development of NAFLD, inflammation and fibrosis (two major factors in the development of NAFLD) have not been sufficiently studied in the present study. Future studies will have to examine the pathogenic mechanisms of NAFLD (i.e., inflammation and fibrosis) in relation to iron overload and changes in acetyl-CoA metabolism.

In conclusion, our study suggests that acetyl-CoA deficiency is involved in the regulation of iron overload on lipid metabolism. Dietary iron overload might slow down the development of NAFLD in ApoE KO mice, possibly due to the shortage of the substrate of cholesterol biosynthesis, i.e., acetyl-CoA. Indeed, iron overload-induced hepatic oxidative damage may impair acetyl-CoA formation from pyruvate and β-oxidation. Dietary iron supplementation could be a potential approach to preventing NAFLD, but additional studies are necessary for confirmation, especially regarding the impact of increased mitochondrial dysfunction and oxidative stress on the liver.

## Figures and Tables

**Table 1:** Quantitative real-time PCR primer sequences.

Gene	Forward Primer 5′–3′	Reverse Primer 5′–3′
Gck	CTGGATGGCTCCGTGTACAAG	CTCCCAGTCATCACGGTCTG
Gapdh	ATACGGCTACAGCAACAGGG	GCTTTGCACATGCCCGGAGCC
Pklr	TGGGAAAACTGGGTGGGATGGATG	GAAGGAAGCAGCCGGGGATTTGAC
Dld	CAGCTCCTTCCTTGTCAACAGA	TTGAACAGAATGGAATAACCGTG
Idh3a	CATCAAGCTCATCACCGAAGAAG	TACAGTTCTCCGCAACTTCCCT
Fh	ATATTGGAGGTGCTACGGAACG	TCCAGTCTGCCAAACCACCA
Mdh2	ATTGCCTCAAAGGTTGTGATGTG	GGATGGTGGAGTTCACTGGGTT
Hmgcr	TGTGGTTTGTGAAGCCGTCAT	TCAACCATAGCTTCCGTAGTTGTC
Hmgcs1	GGGCCAAACGCTCCTCTAAT	AGTCATAGGCATGCTGCATGTG
Hmgcs2	TGCAGGAAACTTCGCTCACA	AAATAGACCTCCAGGGCAAGGA
Sqle	ATAAGAAATGCGGGGATGTCAC	ATATCCGAGAAGGCAGCGAAC
TNF-α	GCATGATCCGCGACGTGGAA	AGATCCATGCCGTTGGCCAG
CRP	ATGGAGAAGCTACTCTGGTGC	ACACACAGTAAAGGTGTTCAGTG
IL-6	TAGTCCTTCCTACCCCAATTTCC	TTGGTCCTTAGCCACTCCTTC
IL-1β	AAACAAAGAAGGCTGGAA	GGTGGCTAAGAACACTGGA
β-actin	TTCGTTGCCGGTCCACACCC	GCTTTGCACATGCCGGAGCC

**Table 2:** Effect of dietary iron overload on biochemical parameters in ApoE KO mice (*n* = 8).

Parameters	Groups		
	ND	HFD	HFD + Fe
Serum iron (mg/L)	193.55 ± 4.65	213.21 ± 6.57	345.70 ± 10.92 ^▲▲^^★★^
Serum TC (mmol/L)	11.62 ± 0.85	20.28 ± 0.67 ^▲▲^	14.63 ± 0.36 ^▲^^★^
Serum LDL-C (mmol/L)	4.95 ± 0.17	11.27 ± 0.52 ^▲▲^	3.20 ± 0.42 ^★★^
Serum AST (U/L)	35.57 ± 5.19	90.19 ± 10.86 ^▲▲^	121.40 ± 12.06 ^▲▲^^★★^
Serum ALT (U/L)	31.88 ± 4.05	60.21 ± 6.05 ^▲▲^	83.97 ± 10.11 ^▲▲^^★^
Hepatic TC (mmol/L)	1.34 ± 0.11	2.94 ± 0.17 ^▲▲^	1.76 ± 0.18 ^★★^
Liver iron (μg/g protein)	234.08 ± 35.25	446.15 ± 20.18 ^▲▲^	1125.23 ± 46.04 ^▲▲^^★★^
Serum hepcidin (ng/mL)	53.62 ± 4.11	60.13 ± 5.03	191.37 ± 11.64 ^▲▲^^★★^

Note: ^▲^ *p* < 0.05 vs. ND; ^▲▲^ *p* < 0.01 vs. ND; ^★^ *p* < 0.05 vs. HFD; ^★★^ *p* < 0.01 vs. HFD. ND, normal diet; HFD, high-fat diet; HFD + Fe, HFD supplied with 2% carbonyl iron; TC, total cholesterol; LDL-C, low-density lipoprotein cholesterol; AST, aspartate aminotransferase; ALT, alanine aminotransferase.

## Data Availability

The data supporting the present findings are contained within the manuscript.

## Supplementary Materials

The following supporting information can be downloaded at: https://www.mdpi.com/article/10.3390/molecules27154966/s1, Figure S1: Western blot analysis of ferritin heavy (H) and light (L) chains in each group; Table S1:The hepatic proteomics analysis data.

## Sample Availability

Samples of the compounds are not available from the authors.
