# Supplementary material for: Acetyl-CoA Deficiency Is Involved in the Regulation of Iron Overload on Lipid Metabolism in Apolipoprotein E Knockout Mice
Source: Molecules. 2022 Aug 4;27(15):4966. doi: 10.3390/molecules27154966 (PMC9370536; doi:10.3390/molecules27154966)
Supplement: Supplementary file 1 [file molecules-27-04966-s001.zip › Figure S1 Western blot analysis of ferritin heavy (H) and light (L) chains in each group.pdf]

Supplemental Figure S1

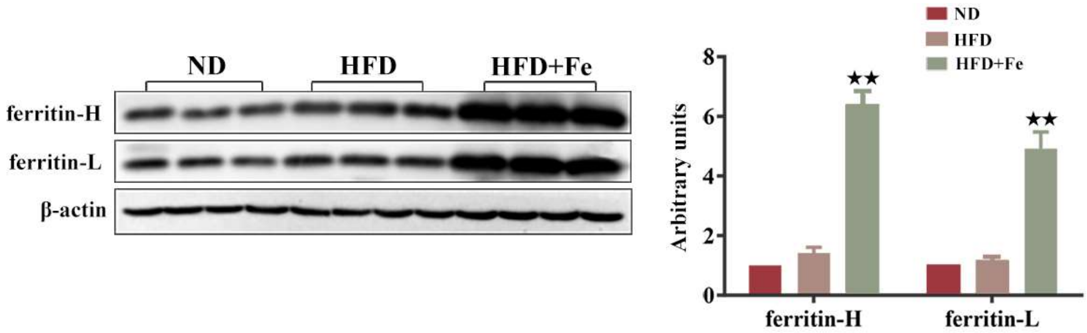

Supplementary Figure S1. Western blot analysis of ferritin heavy (H) and light (L) chains in the normal chow diet (ND), high-fat diet (HFD), or HFD supplemented with 2% carbonyl iron (HFD + Fe) groups. ** p <0.01 vs. the ND and HFD groups.
